# Acetylation of woody lignocellulose: significance and regulation

**DOI:** 10.3389/fpls.2013.00118

**Published:** 2013-05-21

**Authors:** Prashant Mohan-Anupama Pawar, Sanna Koutaniemi, Maija Tenkanen, Ewa J. Mellerowicz

**Affiliations:** ^1^Department of Forest Genetics and Plant Physiology, Swedish University of Agricultural SciencesUmea, Sweden; ^2^Department of Food and Environmental Sciences, University of HelsinkiHelsinki, Finland

**Keywords:** cell wall, wood, biofuel, saccharification, *O*-acetylation, hemicellulose, acetyl esterase

## Abstract

Non-cellulosic cell wall polysaccharides constitute approximately one quarter of usable biomass for human exploitation. In contrast to cellulose, these components are usually substituted by *O*-acetyl groups, which affect their properties and interactions with other polymers, thus affecting their solubility and extractability. However, details of these interactions are still largely obscure. Moreover, polysaccharide hydrolysis to constituent monosaccharides is hampered by the presence of *O*-acetyl groups, necessitating either enzymatic (esterase) or chemical de-acetylation, increasing the costs and chemical consumption. Reduction of polysaccharide acetyl content *in planta* is a way to modify lignocellulose toward improved saccharification. In this review we: (1) summarize literature on lignocellulose acetylation in different tree species, (2) present data and current hypotheses concerning the role of *O*-acetylation in determining woody lignocellulose properties, (3) describe plant proteins involved in lignocellulose *O*-acetylation, (4) give examples of microbial enzymes capable to de-acetylate lignocellulose, and (5) discuss prospects for exploiting these enzymes *in planta* to modify xylan acetylation.

## OCCURRENCE OF *O*-ACETYLATION IN LIGNOCELLULOSE

**FIGURE 1:**
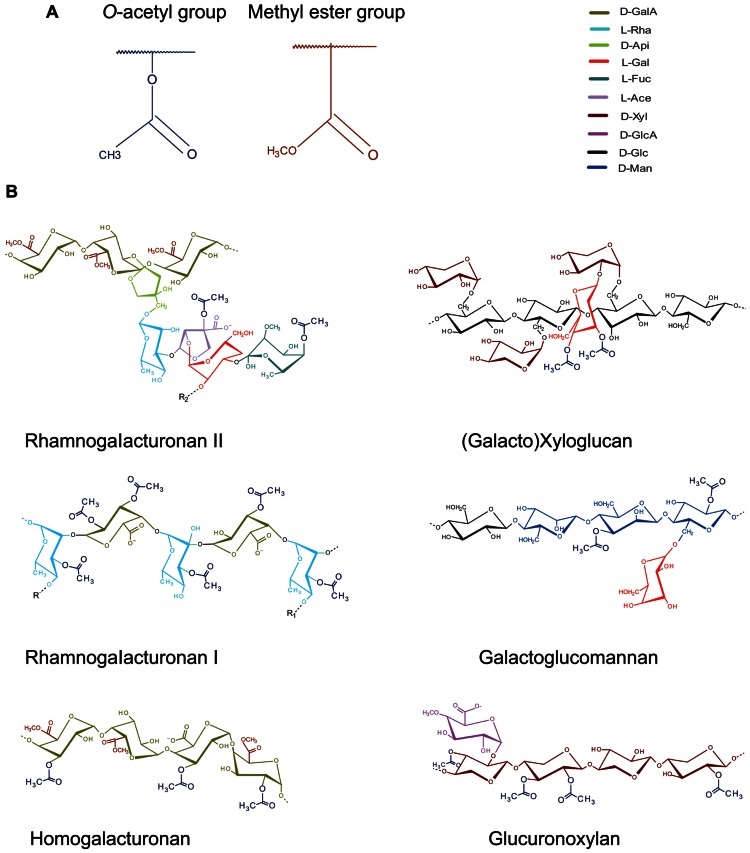
***O*-acetylation of cell wall polysaccharides**. **(A)** Generic representation of *O*-acetyl group as found at different -OH positions in many cell wall polysaccharides. Note the structural similarity between O-acetyl- and methyl ester-groups that decorate carboxylic acid residues in polygalacturonic acid. **(B)** Occurrence of *O-* acetyl groups in cell wall matrix polysaccharides (Voragen et al., 1986; Pauly and Scheller, 2000; MacKinnon et al., 2002; Teleman et al., 2002; Glushka et al., 2003; O’Neill et al., 2004; Ralet et al., 2005).

*O*-acetyl and methyl esterification are the most common substitutions in different cell wall matrix polysaccharides (**Figure 1**). While the role of methyl esterification in plant cell walls has been a focus of many studies, that of *O*-acetylation has received much less attention in the past. *O*-acetylation may occur on the backbones or branches of many cell wall polymers (recently reviewed by Gille and Pauly, 2012), but the nature of acetylated polymer and the extent of acetylation differ between species, tissues and types of cell walls (**Figure 1**; **Table 1**)

**Table 1:** Acetyl contents in lignocellulose from some common woody lignocelluloses in comparison to wheat straw.

Species	% d.w.	Reference
Wheat (straw)	2.2	Sumi (1964)
*Populus tremuloides* Michx. (wood)	3.7	Timell (1967); Sjöström (1993)
*Eucalyptus globulus* Labill. (wood)	3.5	Cetinkol et al. (2010)
*Fagus. grandifolia* Ehrh. (wood)	3.9	Timell (1967)
*Betula papyrifera* Marsh. (wood)	4.4	Timell (1967)
*Picea glauca* Moench. (wood)	1.3	Laffend (1967); Timell (1967)
*Pinus strobus* L. (wood)	1.2	Timell (1967)
*Abies balsamea* Mill. (wood)	1.5	Timell (1967)

In the type I primary walls of softwoods and hardwoods, pectins [homogalacturonan (HG), rhamnogalacturonan I (RGI), and rhamnogalacturonan II] and xyloglucan (XG) are the main sources of *O*-acetyl groups, whereas in the type II primary walls of grasses, the main *O*-acetylated polymer is glucuronoarabinoxylan. The largest pool of acetyl residues in lignocellulose, however, comes from the secondary cell walls since they constitute the bulk of biomass. *O*-acetylation in glucuronoxylan and glucomannan, the main hemicelluloses in secondary cell walls of hardwoods and softwoods, respectively, is found on the *O*-2 and/or *O*-3 positions of the backbone xylopyranosyl or mannopyranosyl residues (Timell, 1967; Teleman et al., 2000; Capek et al., 2002; Willför et al., 2003; Gonçalves et al., 2008; Naran et al., 2009). The minor hemicelluloses are also xylan or mannan-based but more highly branched polymers: galactoglucomannan in hardwoods and glucuronoarabinoxylan in softwoods. Only the mannan is acetylated, suggesting that the function of xylan differs between softwoods and hardwoods.

Typical positions of acetyl groups in these polymers are shown in **Figure 1**, but species-specific positions also may exist (Hayashi, 1989; Pauly, 1999; Jacobs et al., 2002; Jia et al., 2005), and spontaneous migration of acetyl group between the neighboring free hydroxyls is possible (Kabel et al., 2003; Mastihubová and Biely, 2004). Acetylation level is described as degree of acetylation (DA), which is the molecular ratio between the total content of acetyl groups and the total content of monomers that can bear them. DA varied from 0.60 to 0.75 in aspen wood glucuronoxylan and from 0.3 to 0.4 in (galacto)glucomannan in the wood of aspen, birch and spruce (Teleman et al., 2000, 2003). Therefore the overall acetyl content is lower in softwoods than in hardwoods. Existence of specific domains with respect to acetylation, as known for pectin methyl esterification, is an intriguing possibility (Jacobs et al., 2002; Ralet et al., 2008).

Acetyl groups are also found in lignin, linked to the gamma-carbon of the aliphatic side chain of lignin S and G monomers and can be very variable (Del Rio et al., 2007). The highest levels, up to DA 0.8 of S monomers, are found in extraxylary fibers in jute, abaca, and kenaf. In hardwood xylem, lignin acetylation varies between 1 and 50% (w/w) whereas in softwood xylem it has not been reported. The function and consequences of such variability in lignin acetylation are unknown.

## LIGNOCELLULOSE PROPERTIES AFFECTED BY POLYSACCHARIDE ACETYLATION

### POLYMER ACETYLATION AFFECTS ITS INTERACTIONS WITH POLAR MOLECULES

Lignocellulose polysaccharides can be de-acetylated by alkali and re-acetylated by acetic anhydride, providing materials for studying of physico-chemical properties affected by acetylation. Such comparisons show that de-acetylated xylan absorbs more moisture than highly acetylated xylan because it offers more hydrogen bonding to water molecules (Grondahl et al., 2003). The weakly acetylated xylan (DA ~0.5) is totally soluble in water, whereas the totally acetylated xylan (DA 2.0) only dissolves in non-polar solvents like chloroform or polar aprotic solvents like dimethyl sulfoxide. The non-acetylated xylan (DA 0) is only partially soluble in hot water, due to spontaneous intra-molecular hydrogen bonding. De-acetylation of xylan also facilitates its bonding to cellulose (Kabel et al., 2007), whereas its acetylation could be a mean of increasing its interaction with hydrophobic substances like plastic used for making composite wood-based products (Lisperguer et al., 2007) or naturally occurring lignin.

### WOODY BIOMASS DE-ACETYLATION IS IMPORTANT FOR PULPING, SACCHARIFICATION AND FERMENTATION

Wood is de-acetylated during the initial phases of chemical pulping, which consumes most of alkali during Kraft cooking (Zanuttini et al., 2003), and results in the accumulation of acetate in the spent liquor (Sjöström, 1993). Following de-acetylation, fibers swell, which improves their ion transport capacity and facilitates pulping (Sumi, 1964). In mechanical pulping, de-acetylation takes place after the refining step during the alkaline peroxide bleaching. In both pulping processes, de-acetylation of hemicelluloses improves their adsorption to cellulose, which in turn increases the yield and the tensile strength of paper (Laffend, 1967; Zanuttini et al., 2005; Konn et al., 2006).

To convert woody biomass to biofuels, such as bioethanol or biogas, the polysaccharides need to be first hydrolysed to monosaccharides, which are subsequently fermented to ethanol or methane. During saccharification, acetyl groups in xylans and mannans create steric hindrance for binding of many hydrolytic enzymes, which limits the extent of hydrolysis (recently reviewed by Biely (2012). For example, the action of endoxylanases is partially or completely hindered by acetyl groups (Biely et al., 1986; Grohmann et al., 1989). Sugar yields of β-xylosidases, β-mannosidases, and β-glucosidases are increased by the addition of suitable esterases, indicating that these hydrolytic enzymes cannot release acetylated terminal residues from hemicellulosic oligosaccharides. Acetylation of xylan also limits its hydrolysis by acid (Chen et al., 2012). De-acetylation of hemicelluloses is therefore a prerequisite for their saccharification, which in turn is important for opening cellulose surface to cellulolytic enzymes (Vazquez et al., 2001; Selig et al., 2009; Zhang et al., 2011).

Chemical de-acetylation of wood is stoichiometric, thus 100 g of aspen wood requires ~4 g of KOH for complete de-acetylation (Kong et al., 1992). Diluted alkali removed acetyl esters without affecting lignin or xylan, which increased total monosaccharide yield approximately fourfold. In similar experiments with American aspen, up to 90% de-acetylation improved cellulose and xylan conversion two and sevenfold, respectively (Holtzapple and Chang, 2000). Chemical de-acetylation, which is done prior the enzymatic hydrolysis, lowers the solubility and extractability of xylans and mannans limiting their hydrolysis (Tenkanen, 1995). In contrast, the enzymatic de-acetylation does not induce such undesirable changes since it is carried out simultaneously with saccharification.

De-acetylation of lignocellulose results in the accumulation of acetate in the medium, which depending on the pH, might be in a protonated form. Small amounts of acetic acid stimulate the metabolism of common yeast-strains (Maiorella et al., 1983; Almeida et al., 2007), but higher concentrations, starting at the levels typical for softwood processing, inhibit yeast growth and fermentation (Olsson and Hahn-Hägerdal, 1996; Ranatunga et al., 1997; Helle et al., 2003).

### ACETYLATION OF SECONDARY BUT NOT PRIMARY WALLS INCREASES MECHANICAL STRENGTH

While the high hemicellulose acetylation is disadvantageous for pulping and biofuel production, it is often desirable in solid wood products. Wood acetic anhydride treatment, resulting in ~5–15% of weight gain, increases wood mechanical strength (modulus of elasticity and rupture) in both tension and compression experiments, but higher levels of acetylation are damaging (Ramsden et al., 1997; Papadopoulos and Pougioula, 2010). Interestingly, the acetylation is initially introduced to the secondary wall layers where hydroxyl groups of hemicelluloses are likely the main reactants, whereas prolonged treatment introduces acetyl to the middle lamella where pectins and lignin are the main targets (Rowell, 2009). Most likely it is the acetylation of xylan and mannan in secondary wall layers that is responsible for the increased stiffness, possibly by allowing more hydrophobic interactions with lignin.

Such a mechanism is not possible in non-lignified primary walls. Indeed, it has been shown that overexpression of pectin acetyl esterase (PAE) inhibited cell elongation in tobacco (Gou et al., 2012) and led to stiffer cell walls in potato tubers based on mechanical stress/strain experiments (Orfila et al., 2012). Moreover, primary cell wall acetylation was negatively correlated with cell adhesion (Liners et al., 1994). Since pectin acetylation, similarly, to methyl esterification, interferes with binding of calcium to polygalacturonic acid and formation of “egg-box” domains in cell wall (Ralet et al., 2003), pectin acetylation decreases cell wall stiffness.

### ACETYLATION AFFECTS WOOD BIOTIC RESISTANCE

Since acetylation of xylan and mannan hinders their hydrolysis, chemical acetylation of wood has been used to increase its durability and resistance to fungi, bacteria, and termites (Peterson and Thomas, 1978; Mohebby, 2003; Rowell, 2009). It was therefore surprising to find that reduced acetylation in different polymers, XG and pectins – in *rwa2* mutants, xylan – in lines overexpressing acetyl xylan esterase (AXE) from family carbohydrate esterase 1 (CE1), and pectin – in lines overexpressing rhamnogalacturonan acetyl esterase (RGAE), induced resistance to necrotrophic fungi (Manabe et al., 2011; Pogorelko et al., 2013). Moreover, whereas digestibility of pectins by *Aspergillus *pectinase was actually reduced by their de-acetylation (Gou et al., 2012), digestibility of cell walls of plants expressing either PAE or RGAE by pectinase/PME mixture was increased (Orfila et al., 2012; Pogorelko et al., 2013). Overexpressed esterases were shown to activate plant acetylation and defense pathways, and it has been proposed that the cell wall fragments generated as a result of de-acetylation may trigger the activation of plant innate immune responses (Pogorelko et al., 2013). Clearly, more studies are needed to understand how acetylation of different polymers affects their digestibilities *in vivo* and *in vitro* by different hydrolases to gain understanding of the role of their acetylation in biotic stress resistance.

## ENZYMES DE-ACETYLATING LIGNOCELLULOSE POLYSACCHARIDES

### DE-ACETYLATION OF XYLAN AND MANNAN

Polymeric xylan and xylo-oligosaccharides are de-acetylated by AXEs (EC 3.1.1.72). Short xylo-oligosaccharides can be also de-acetylated by non-specific acetyl esterases (AE; EC 3.1.1.6), which act mainly on the non-reducing end residues (Poutanen et al., 1990; Linden et al., 1994). AXEs and AEs have been found in wood-degrading fungi and bacteria (Biely et al., 1985; Dupont et al., 1996; Biely, 2012). The occurrence of *true* AXEs in plants has not been reported, although poplar PAE1 had some activity toward acetylated xylan (Gou et al., 2012).

Acetyl xylan esterases fall presently into eight of the 16 CE families (http://www.cazy.org/), including CE1–CE7, and CE16 (**Table 2**; Dodd and Cann, 2009; Biely, 2012; Gou et al., 2012). Most CE1–CE7 enzymes are serine esterases having Ser-His-Asp(Glu) triad or Ser-His diad in their active sites and use the catalytic mechanism with the formation of enzyme-Ser complex (acetylation), followed by the de-acetylation by activated water molecule. CE4 enzymes have a unique, Asp-His and divalent cation-dependent activity (Taylor et al., 2006; Biely, 2012).

**Table 2:** Examples of enzymes deacetylating plant cell wall poly and oligosaccharides.

CAZY	Species	Activity^1^	Reference	pH	Protein name(s)	Accession number
CE1	*Aspergillus awamori*	AXE****	Koseki et al. (2005)	6–7	AXEA	BAA13434
	*Aspergillus oryzae*	AXE	Koseki et al. (2006)	6–7	AXE	BAD12626
	*Aspergillus niger*	AXE	Kormelink et al. (1993)	5.5	AXEA	CAK46215
	*Penicillium purpurogenum*	AXE	Gordillo et al. (2006)	6	AXEI	AAM93261
CE2	*Neocallimastix patriciarum*	AXE	Dalrymple et al. (1997)	7	BNAI, BNAA	AAB69090
	*Cellvibrio japonicus*	AXE and AGME	Montanier et al. (2009)	7	AXE2B, CE2C	ACE85140
CE3	*Clostridium thermocellum*	AXE	Correia et al. (2008)	7	CES3	ABN52033
CE4	*Streptomyces lividans*	AXE	Dupont et al. (1996)	6–7	AXEA	AAC06115
CE5	*Trichoderma reesei *	AXE	Sundberg and Poutanen (1991); Margolles-Clark et al. (1996)	5–6	AXE	Z69256
	*Penicillium purpurogenum *	AXE	Egaña et al. (1996)	6	AXEII	AAC39371
CE6	*Fibrobacter succinogenes*	AXE	Yoshida et al. (2010)	7.5	AXE6A	AF180369
CE7	*Thermoanaerobacterium*	AXE and CCD	Shao and Wiegel (1995)	6	AXE1	AF001926
	*Bacillus pumilus*	AXE and CCD	Degrassi et al. (2000)	7	AXE	AJ249957
CE10	*Erwinia chrysanthemi*	PAE	Shevchik and Hugouvieux-Cotte-Pattat (1997)	8	PAEY	CAA70971
	*Erwinia chrysanthemi*	PAE enhanced by PEL	Shevchik and Hugouvieux-Cotte-Pattat (2003)	8.5	PAEX	CAD45188
CE12	*Bacillus subtilis*	RGAE, CCD, and AXE enhanced by Xyn10	Martinez-Martinez et al. (2008)	8.5	YEST	CAB12521
CE13	*Populus trichocarpa*	PAE and AXE	Gou et al. (2012)	7.0	PAE1, CE13_5	HQ223420
CE16	*Trichoderma reesei*	AE enhanced by xylanases and mannanases	Poutanen et al. (1990); Li et al. (2008)	5.5	AES1	ABI34466

1 AE, acetyl esterases (AE; EC 3.1.1.6); AGME, acetyl glucomannan esterase (EC 3.1.1.- ); AXE, acetyl xylan esterase (EC 3.1.1.72); CCD, cephalosporin C deacetylase (EC 3.1.1.41); RGAE, rhamnogalacturonan acetyl esterase (EC 3.1.1.86); PAE, pectin acetyl esterase (EC 3.1.1.-); PEL, pectate lyase (EC 4.2.2.2).

Different AEs and AXEs may exhibit preferences to different acetyl positions (Christov and Prior, 1993; Linden et al., 1994; Biely, 2012). For example, CE1, CE4, and CE5 AXEs have preference for position *O*-2, CE16 AEs for positions *O*-3 and *O*-4 (Biely et al., 2011) and CE2 AXEs for position *O*-6 in hexoses (Topakas et al., 2010). Many CE1 and CE2 AXEs have broad specificities for xylan and mannan. Acetyl glucomannan esterase (AGME, EC 3.1.1.- ) activity was shown in *Aspergillus sp.*, and the enzyme was also capable of slow de-acetylation of xylan (Tenkanen et al., 1995). CE family for this enzyme remains to be identified.

### DE-ACETYLATION OF PECTINS

Pectin acetyl esterases (EC 3.1.1.-) were found in plant and microbial species (Williamson, 1991; Breton et al., 1996; Shevchik and Hugouvieux-Cotte-Pattat, 1997, 2003; Gou et al., 2012). Plant PAEs belong to family CE13 and are secreted enzymes acting on *O*-2 and *O*-3 acetyl in HG. *Arabidopsis* and *Populus* have 12 and 9 CE13 members, respectively (Geisler-Lee et al., 2006). Genomic sequencing identified similar proteins in animals and bacteria, but corresponding activities have not been characterized. Bacterial PAEs of *Erwinia chrysanthemi* PaeX and PaeY, acting on demethylated oligomeric and polymeric HG, respectively, are classified in CE10 (Shevchik and Hugouvieux-Cotte-Pattat, 1997, 2003).

Rhamnogalacturonan acetyl esterase (EC 3.1.1.86) de-acetylates RGI at GalA *O-*2 and *O-*3 positions and belongs to CE12 (Molgaard et al., 2000). This activity has been shown in *Aspergillus aculeatus *(Schols et al., 1990), and in bacteria where it has broad substrate specificity including acetylated xylan and cephalosporin C (Martinez-Martinez et al., 2008; Navarro-Fernandez et al., 2008).

## BIOSYNTHESIS OF ACETYLATED POLYSACCHARIDES IN PLANTS

*O*-acetylation of plant cell wall-polysaccharides takes place in the Golgi. In the case of HG, RGI, and XG, acetyl-CoA has been identified as a donor substrate (Pauly and Scheller, 2000). Proteins involved in polysaccharide acetylation are conserved in pro- and eukaryotes (Gille and Pauly, 2012). In fungi, animals and Gram-positive bacteria, the acetyl transfer to extra-cytoplasmic compartment and catalysis are performed by a single multifunctional protein Cas1p identified first in *Cryptococcus neoformans*. Cas1p has a set of 12 transmembrane domains (called Cas1p domain) that are proposed to form a channel for acetyl-CoA transfer, and two other domains, TRICHOME-BIREFRINGENCE-LIKE (TBL)-domain and DUF231 located at the extra-cytoplasmic side, that are involved in esterification and are conserved in serine esterases/lipases of SGNH superfamily including AXEs (Dodd and Cann, 2009).

In plants, two separate gene families are needed for acetylation of cell wall polymers. REDUCED WALL ACETYLATION (RWA) family, which has the Casp1 domain (Lee et al., 2011; Manabe et al., 2011), and TBL family, which has TBL and DUF231 domains (Anantharaman and Aravind, 2010; Bischoff et al., 2010a,b; Gille et al., 2011a).

*Arabidopsis* RWA family has four members. RWA1, RWA3, and RWA4 were suggested to redundantly regulate acetylation in secondary walls (Lee et al., 2011) whereas RWA2 was shown to be responsible for acetylation of XG and pectin (Manabe et al., 2011). Quadruple *rwa1/2/3/4* mutants show 42% loss of acetyl groups in xylan and 40% reduction in stem acetyl content (Lee et al., 2011). These results indicate that RWA regulates acetylation in several polymers and is partially redundant with some other presently unknown proteins. *Arabidopsis* TBL family has 45 members (Anantharaman and Aravind, 2010). Two of them, TBL-27/AXY4 and TBL-22/AXY4L, are required for XG acetylation in vegetative tissues and in seeds, respectively, but do not affect acetylation of pectins, xylan or mannan (Gille et al., 2011a). Deep sequencing *Amorphophallus konjac *corm indicated that proteins similar to AtTBL-25 and AtTBL-26 might be mannan *O*-acetyltransferases (Gille et al., 2011b). Recently, the main putative xylan acetyl transferase has been identified as AtTBL-29/ESKIMO1 (Xiong et al., 2013). The *eskimo1* mutants had 60% reduced acetylation of xylan and a smaller reduction in mannan acetylation but pectin or XG acetyl content was not affected. These results support the proposal that the TBL-family members encode acetyl transferases acting on specific polymers (Gille et al., 2011a; Gille and Pauly, 2012).

## PROSPECTS FOR MODIFYING POLYSACCHARIDE *O*-ACETYLATION IN PLANTS

Different roles are emerging for acetylation in different plant polymers, for example regarding mechanical properties of pectin and xylan, as we have discussed here. Genetic tools are now in place to systematically modify acetylation in specific polysaccharides (but not yet in lignin), and to study mechanical properties of such modified plants. These studies, supplemented by *in vitro* analyses of the rheological properties of polymers, would provide a framework for understanding molecular mechanisms operating in cell walls that are affected by polymer acetylation.

Considering the high impact of polysaccharide acetylation for downstream utilization of woody lignocellulose, it appears that DA of different polymers is an important target for the feedstock improvement. Surprisingly, the knowledge of natural variation of these traits in tree species is virtually missing. One major obstacle for gathering such data and including acetylation traits in conventional breeding programs is the shortage of high throughput analytical tools for detailed analysis of degree and position of acetylation in different plant cell wall polysaccharides.

However, genetic engineering of feedstocks with altered acetylation seems feasible in a near future. Based on studies published since 2011, it appears that moderate (by ~20%) reduction of general acetylation levels, *in planta* by mutating biosynthetic genes (Lee et al., 2011; Manabe et al., 2011) or by introducing an AXE to the apoplast for post-synthetic acetyl removal (Pogorelko et al., 2011) is tolerated by herbaceous species, however, too strong de-acetylation of xylan might lead to undesirable molecular changes in cell wall (Poutanen et al., 1990) resulting in growth defects as observed in the *rwa1/2/3/4* and *tbl-29* mutants (Lee et al., 2011; Xiong et al., 2013). Also, post-synthetic de-acetylation of pectins was shown to affect stem and reproductive organ growth (Gou et al., 2012). Thus, the kind of polymer affected, and the degree of de-acetylation matter for plant performance and might need to be optimized. Increased acetylation* in planta*, which might be desirable in solid wood products, has not been so far demonstrated. The overexpression of TBL-29 did not result in higher acetyl content in *Arabidopsis* (Xiong et al., 2013). Thus it is not yet known if increase of cell wall acetylation can be obtained and tolerated by plants.

Little is known about the performance of acetyl-modified plants in various applications or about the goals for acetyl optimisation. For example, extractability of polymers is likely a key parameter that is affected by acetylation, and has not received much attention. Saccharification is another matter – although xylan acetylation restricts its hydrolysis, opposite results have been obtained with pectin (Gou et al., 2012). Only a few reports exist on acetyl-modified plants where the cell wall context and type of pretreatment come into play: saccharification yields of *rwa1/2/3/4* mutants were not increased compared to wild type in tests without pretreatment (Lee et al., 2011) whereas *tbl-29 *mutant showed a 10% decrease in glucose yield per cell wall mass (Xiong et al., 2013). However, taking into account ~20% reduction in cellulose content in *tbl-29* would reveal that a higher proportion of cellulose was hydrolysed in the mutant than in wild type. ~20% reduction in acetyl content in plants overexpressing AXE did not improve saccharification after acid pretreatment (Pogorelko et al., 2011). Clearly, analysis of a range of transgenic lines with different levels of de-acetylation, using standardized protocols is needed to optimize their acetyl level taking into account both plant and lignocellulose performance in a process.

## Conflict of Interest Statement

The authors declare that the research was conducted in the absence of any commercial or financial relationships that could be construed as a potential conflict of interest.
